# Combined Adenovirus-Mediated Artificial microRNAs Targeting mfgl2, mFas, and mTNFR1 Protect against Fulminant Hepatic Failure in Mice

**DOI:** 10.1371/journal.pone.0082330

**Published:** 2013-11-26

**Authors:** Dong Xi, Ming Wang, Huali Ye, Xiaoping Luo, Qin Ning

**Affiliations:** 1 Department and Institute of Infectious Disease, Tongji Hospital, Tongji Medical College, Huazhong University of Science and Technology, Wuhan, China; 2 Department of Pediatrics, Tongji Hospital, Tongji Medical College, Huazhong University of Science and Technology, Wuhan, China; Yonsei University College of Medicine, Korea, Republic Of

## Abstract

Hepatitis B virus (HBV)-related acute-on-chronic liver failure (ACLF) has a poor prognosis with high in-hospital mortality. Hepatic and circulating inflammatory cytokines, such as fibrinogen like protein 2 (fgl2), FasL/Fas, and TNFα/TNFR1, play a significant role in the pathophysiology of ACLF. This study aimed to investigate the therapeutic effect of recombinant adenoviral vectors carrying constructed DNA code for non-native microRNA (miRNA) targeting mouse fgl2 (mfgl2) or both mFas and mTNFR1 on murine hepatitis virus (MHV)-3-induced fulminant hepatitis in BALB/cJ mice. Artificial miRNA eukaryotic expression plasmids against mfgl2, mFas, and mTNFR1 were constructed, and their inhibitory effects on the target genes were confirmed *in vitro*. pcDNA6.2-mFas-mTNFR1- miRNA，which expresses miRNA against both mFas and mTNFR1 simultaneously，was constructed. To construct a miRNA adenovirus expression vector against mfgl2, pcDNA6.2-mfgl2-miRNA was cloned using Gateway technology. Ad-mFas-mTNFR1- miRNA was also constructed by the same procedure. Adenovirus vectors were delivered by tail-vein injection into MHV-3-infected BALB/cJ mice to evaluate the therapeutic effect. 8 of 18 (44.4%) mice recovered from fulminant viral hepatitis in the combined interference group treated with Ad-mfgl2-miRNA and Ad-mFas-mTNFR1-miRNA. But only 4 of 18 (22.2%) mice receiving Ad-mfgl2-miRNA and 3 of 18 (16.7%) mice receiving Ad-mFas-mTNFR1- miRNA survived. These adenovirus vectors significantly ameliorated inflammatory infiltration, fibrin deposition, hepatocyte necrosis and apoptosis, and prolonged survival time. Our data illustrated that combined interference using adenovirus-mediated artificial miRNAs targeting mfgl2, mFas, and mTNFR1 might have significant therapeutic potential for the treatment of fulminant hepatitis.

## Introduction

Chronic hepatitis B virus (HBV) infection is an important global health problem with approximately 350 million individuals worldwide infected with chronic HBV [1], and in Asia and Africa there is a particular high prevalence of chronic HBV infection, where the chronic carrier rate is (10~20)% [2,3]. Acute-on-chronic liver failure (ACLF) is mainly due to viral infection, primarily hepatitis B, particularly in Asia where HBV-ACLF accounts for more than 80% of all ACLF cases [4,5]. Current treatment strategies for ACLF include liver support systems [6] and internal treatments, which have met with only partial success. The mortality from ACLF is greater than 80% in cases without emergency liver transplantation [7,8]. 

The lack of effective therapeutic options for patients with HBV-ACLF results in immediate deterioration and a subsequent poor prognosis. Therefore, it is necessary to develop more effective therapies that can effectively decrease the mortality of ACLF with fewer side effects. The immune-mediated liver injury is considered to play a pivotal role in the progress from chronic HBV infection to ACLF. Hepatic and circulating inflammatory cytokines play a significant role in the pathophysiology of ACLF, including hepatocyte necrosis and apoptosis. On one hand, activation of the coagulation cascade is an integral component of host inflammation. Fibrin deposition and thrombosis within the microvasculature is now appreciated as playing a vital role in the hepatocellular injury observed during severe viral hepatitis [9-12]. We constructed an mfgl2 antisense plasmid and found that gene silencing of the fgl2 gene significantly inhibited mfgl2 expression and ameliorated murine hepatitis virus strain 3 (MHV-3)–induced fulminant hepatitis in BALB/cJ mice [10]. On the other hand, hepatocyte apoptosis is a key feature of virtually all acute and chronic liver diseases, including acute liver failure (ALF). The Fas ligand (FasL)/Fas and TNFα/TNF type-I receptor (TNFR1) apoptotic pathways are the two major mechanisms regulating this phenomenon. Song et al. used small interfering RNAs (siRNAs) targeting the Fas gene to significantly increase the 10-day survival rate in mice with agonistic Fas-specific antibody-induced fulminant hepatitis [13]. Jiang et al. further demonstrate that galactose-conjugated liposome nano-particles bearing Fas siRNA prevent ConA-induced fulminant hepatitis [14].

Over the years, adenovirus continues to occupy center stage in gene therapy as a vector for transgene delivery. Adenoviral gene transfer of NF-κB inhibitory protein ABIN-1 protected mice from TNF/Galactosamine-induced acute liver failure and lethality[15]. Adenovirus encoding CTLA-4Ig, a fusion protein consisting of the extracellular domain of mouse cytotoxic T-lymphocyte antigen-4 (CTLA-4) and a human immunoglobulin (Ig) Fc fragment, suppressed liver injury by blockade of costimulatory signals in a mouse model of fulminant hepatitis induced by injection of *Propionibacterium acnes* and lipopolysaccharide[16]. And microRNAs (miRNAs) are small, noncoding RNAs of 21~24 nucleotides that function to negatively regulate gene expression at a post-transcriptional level [17,18]. Adenoviral-based delivery of DNA for a non-native miRNA to limit RNA translation in heart demonstrates significant and acute silencing of target RNA *in vivo*[19]. To date, there have been no reports on the inhibition of the gene expression of immune-related factors such as fgl2, Fas, and TNFR1 utilizing an adenovirus miRNA-based system.

In the present study, we constructed recombinant adenoviral vectors carrying DNA code for non-native miRNAs that targeted mfgl2 (Ad-mfgl2-miRNA) or targeted both mFas and mTNFR1 (Ad-mFas-mTNFR1-miRNA), and we sought to evaluate the therapeutic effect of these adenoviral vectors by single or combined use *in vivo*.

Materials and Methods

Virus

MHV-3 was obtained from the American Type Culture Collection (ATCC, Rockville, MD, USA), plaque purified on monolayers of DBT (delayed brain tumor) cells (ATCC, Manassas, VA, USA), and titered on L2 cells (ATCC, Manassas, VA, USA) according to a standard plaque assay [20]. The titers were 2×10^7^ plaque-forming units (PFU)/mL. Virus was harvested by centrifugation at 4500 g for 1 h at 4 °C. 

### Animal model

Eight- to-ten-week-old female BALB/cJ mice (weighing 20-22 g) were purchased from the Laboratory Animal Center of Wuhan University (Wuhan, China), and all mice were maintained under specific pathogen-free (SPF) and controlled conditions at the Institute of Infectious Disease of Tongji Hospital (Wuhan, China). All animal experiments were carried out according to the guidelines of the Chinese Council on Animal Care and approved by Tongji Hospital Committees on Animal Experiment. The research protocol was reviewed and approved by the hospital institutional review board of Tongji Hospital. MHV-3 was reconstituted in sterile phosphate-buffered saline (PBS) at a concentration of 100 PFU/ml. 0.5 ml of 4×10^8^ PFU/ml Ad-mfgl2-miRNA, Ad-mFas-mTNFR1-miRNA, or an irrelevant sequence control, was injected via the tail vein for each mouse(18 animals in each group，for combination interference, both 0.25 ml of 4×10^8^ PFU/ml Ad-mfgl2-miRNA and 0.25 ml of 4×10^8^ PFU/ml Ad-mFas-mTNFR1-miRNA for each mouse). At 24 h, mice received 20 PFU of MHV-3 intraperitoneally to develop fulminant viral hepatitis. Liver sections and serum samples from separate sets of animals were sampled at the indicated times after MHV-3 infection.

### Construction of miRNA expression plasmids

According to their cDNA sequences on PubMed (mfgl2: NM_008013, mFas: NM_007987, mTNFR1: NM_0116094), three pairs of single-stranded complementary miRNA oligonucleotides against mfgl2, mFas, and mTNFR1 (named mfgl2-miRNA, mFas-miRNA, and mTNFR1-miRNA, respectively) were designed using miRNA software from Invitrogen (Carlsbad, CA, USA) and then synthesized. The double-stranded miRNA templates for mfgl2, mFas, and mTNFR1 genes were inserted into the miRNA expression plasmid pcDNA-6.2-GW/EmGFP-miR (Invitrogen, Carlsbad, CA, USA) using T4 DNA ligase to construct the three plasmids: pcDNA6.2-mfgl2-miRNA, pcDNA6.2-mFas-miRNA, and pcDNA6.2-mTNFR1-miRNA, respectively. Meanwhile, an irrelevant pre-miRNA sequence(named Neg-miRNA) provided by Invitrogen (Carlsbad, CA, USA) was also cloned. Sequencing and screening for miRNA expression plasmids with no mutations was performed. The oligonucleotide sequences are shown below:

mfgl2-miRNA: top: 5’-TGCTGTTGGCTGGGACACTTGGAACAGTTTTGGCCA
CTGACTGACTGTTCCAAGTCCCAG-3’, bottom: 5’-CCTGTTGGCTGGGACTT
GGAACAGTCAGTCAGTGGCCAAAACTGTTCCAAGTGTCCCAGCCAAC-3’;
mFas-miRNA: top: 5’-TGCTGTCAGGGTGCAGTTTGTTTCCAGTTTTGGCCA
CTGACTGACTGGAAA-3’, bottom: 5’-CCTGTCAGGGTGCAGTGTTTCCAGTC
AGTCAGTGGCCAAAACTGGAAACAAACTGCACCCTGAC-3’; mTNFR1- miRNA:
top: 5’-TGCTGAAGGATAGAAGGCAAAGACCTGTTTTGGCCACTGACTGACTGAC AGGTCTTTCTTCTATCCTT -3’, bottom: 5’-CCTGAAGGATAGAAGAAAGACCTGTCA GTCAGTGGCCAAAACAGGTCTTTGCCTTCTATCCTTC-3’; and neg-miRNA:
5’-GAAATGTACTGCGCGTGGAGACGTTTTGGCCACTGACTGACGTCTCCACGCAGTACATTT-3’. A sketch of construction of miRNA expression plasmids was shown in Figure S1.

To construct one expression plasmid that could express miRNA against both mFas and mTNFR1 (named pcDNA6.2-mFas-mTNFR1-miRNA), pcDNA6.2-mFas-miRNA and pcDNA6.2-mTNFR1-miRNA were digested with *Bam*HI and *Xho*I or *Bgl*II and *Xho*I, respectively. After electrophoresis, the short fragment of pcDNA6.2-mFas-miRNA and the long fragment of pcDNA6.2-mTNFR1-miRNA were extracted using a QIAquick Gel Extraction Kit (Qiagen, Hilden, Germany), ligated with T4 DNA ligase, and transfected into *E. coli* TOP10 competent cells (Invitrogen, Carlsbad, CA, USA). A sketch of construction of pcDNA6.2-mFas-mTNFR1-miRNA was shown in Figure S2.

### Generation of miRNA adenoviral expression vectors

To construct miRNA adenovirus expression vectors against mfgl2, mFas, mTNFR1, and the irrelevant sequence, the ViraPower Adenoviral Expression System (Invitrogen, Carlsbad, CA, USA) was used in accordance with the manufacturer’s protocol. As an example, Gateway technology was used to recombine the pcDNA6.2-mfgl2-miRNA plasmid with the pAd/CMV/V5-DEST vectors (Invitrogen, Carlsbad, CA, USA) to form the adenovirus expression vector Ad-mfgl2-miRNA. Ad-mfgl2-miRNA was transformed into *E.Coli* TOP10 competent cells (Invitrogen, Carlsbad, CA, USA), and single clones were isolated and assessed via sequence analysis. Correct constructs were then transfected into 293A cells. After an 80% cytopathic effect (CPE) was observed, adenovirus-containing cells were harvested and crude viral stocks was collected via freeze/thaw cycles followed by centrifugation. The amplified adenovirus was purified using the Adeno-X Virus Purification Kit (Clontech, Mountain View, CA, USA). The titer of adenovirus was determined based on the 50% tissue culture infective dose (TCID_50_). 

### Transfection

Chinese hamster ovary (CHO) cells(ATCC, Manassas, VA, USA) were cultured in six-well plates with DMEM (Gibco BRL, Grand Island, NY, USA) supplement with 10% FBS (Gibco BRL, Grand Island, NY, USA) until 80-90% confluence. 2μg pcDNA6.2-mfgl2-miRNA, pcDNA6.2-mFas-miRNA, or pcDNA6.2-mTNFR1-miRNA was mixed with 2μg pcDNA3.1-mfgl2 (constructed in our lab), pcDNA3.1-mFas, or pEGFP-mTNFR1 (constructed in our lab) in serum-free DMEM, respectively. 2μg pcDNA6.2-mFas-mTNFR1-

miRNA was mixed with 1μg pcDNA3.1-mFas and 1μg pEGFP-mTNFR1 in serum-free DMEM. The mixture was then combined with Lipofectamine 2000 (2.5 μg/μl; Invitrogen, Carlsbad, CA, USA) and gently mixed. After incubation at room temperature for 20 min, the complex solution was added to CHO cells and incubated at 37°C in 5% CO_2_. The medium was replaced with fresh medium with 10% FBS 4~6 h after transfection. At 48 h after transfection, cells were harvested for qRT-PCR and Western blot analysis to evaluate the inhibitory effect of pcDNA6.2-mfgl2-miRNA, pcDNA6.2-mFas-miRNA, or pcDNA6.2-mTNFR1-miRNA. pcDNA6.2-neg-miRNA was used as a negative control.

Mice liver cell line IAR20(ATCC, Manassas, VA, USA) was cultured in six-well plates with DMEM (Gibco BRL, Grand Island, NY, USA) supplement with 10% FBS (Gibco BRL, Grand Island, NY, USA) until 70-80% confluence. 3μg pcDNA6.2-mFas-miRNA or pcDNA6.2-mTNFR1-miRNA was mixed with 3μg pcDNA3.1-mFas or pEGFP-mTNFR1 in 300μl serum-free DMEM, respectively. 3μg pcDNA6.2-mFas-mTNFR1-miRNA was mixed with 1.5μg pcDNA3.1-mFas and 1.5μg pEGFP-mTNFR1 in serum-free DMEM. The mixture was then combined with 50μl PolyFect Transfection Reagent (Qiagen, Hilden, Germany) and gently mixed. After incubation at room temperature for 5 min, the complex solution was added to IAR20 cell and incubated at 37°C in 5% CO_2_. The medium was replaced with fresh medium with 10% FBS 8 h after transfection. At 48 h after transfection, cells were harvested for qRT-PCR and Western blot analysis to evaluate the inhibitory effect of pcDNA6.2-mFas-miRNA, pcDNA6.2-mTNFR1-miRNA or pcDNA6.2-mFas-mTNFR1-miRNA.

### Real-time fluorescence quantitative RT-PCR

Total RNA was extracted from mouse liver using TRIzol reagent (Invitrogen, Carlsbad, CA, USA) according to protocol provided by the manufacturer and equal quantities of RNA were then reverse transcribed into cDNA (Fermentas, Burlington, ON, Canada). Equal quantities of cDNA from each animal were used for real-time RT-PCR. Fluorescence quantitative RT-PCR was done using SYBR Green reagents (Toyobo, Tokyo, Japan) with an ABI PRISM 7500 sequence detection system (Applied Biosystems, Foster City, CA, USA) to detect RNA expression levels. The relative expression levels for each target gene were normalized to GAPDH and are presented as fold increases relative to sham mice. Quantification was performed according to the comparative Ct method (△△C_T_). Results are expressed relative to GAPDH values. All reactions were carried out in a 20 μl volume. The specific primers used in this experiment were as follows:

mfgl2 sense, 5’-TCTGGGAACTGTGGGCTCTATT-3’; mfgl2 antisense, 5’-GGACACCTTTGTATTTCTGGTGGTA-3’; mFas sense, 5’-ACCTCCAGTCGTG-AAACC-3’; mFas antisense, 5’-CATCTATCTTGCCCTCCT-3’; mTNFR1 sense, 5’-GAGGACCGTACCCTGATC-3’; mTNFR1 antisense, 5’-GACAACGCTCGTGA-ATGA-3’; MHV-3 sense, 5’-TTCCTACTTTGCGCCC-3’; MHV-3 antisense, 5’-TATTTGGCCCACGGGATTGT-3’; GAPDH sense, 5’-CGGATTTGGTCGTATTGG-3’; and GAPDH antisense, 5’-CTCGCTCCTGGAAGATGG-3’. Target gene expression at the RNA level was normalized to GAPDH. PCR was performed by initial denaturation at 95°C for 5 minutes, then denaturation at 95°C for 30 seconds, annealing at 50°C for 5 seconds, and extension at 72°C for 15 seconds for a total of 40 cycles. 

### Serum aminotransferase measurements

Serum alanine aminotransferase (ALT) levels were measured using a clinical chemistry analyzer (Aeroset system; Abbott Laboratories, Abbott Park, IL, USA).

### Immunohistochemical staining

Liver tissues were isolated, sliced, fixed, dehydrated, and then subjected to immunohistochemistry using PV6001 PowerVision Two-Step Histostaining Reagent (ZSGB-BIO, Beijing, China) according to the manufacturer’s instructions. Liver tissue sections were incubated with a rabbit polyclonal antibody against mfgl2 prothrombinase (1:200 dilution; Abcam, Cambridge, UK), mFas (1:50 dilution; Abcam, Cambridge, UK), and mTNFR1 (1:1000 dilution; Abcam, Cambridge, UK) or with a polyclonal antibody against fibrinogen (1:1000 dilution, Dako, Glostrup, Denmark) as previously described [9]. After incubation with horseradish peroxidase (HRP)-labeled goat IgG fraction to rabbit IgG Fc, the target protein was detected using a diaminobenzidine (DAB) kit (ZSGB-BIO, Beijing, China). The slides were then counterstained with hematoxylin and visualized using a microscope(Olympus, Tokyo, Japan). 

### Protein preparation

Mitochondria were isolated with a tissue mitochondria isolation kit （Beyotime Biotechnology, Haimen, China) according to the manufacturer’s instructions. During protein preparation, all samples were placed on ice. Seventy-five mg liver tissue was cut into pieces, tissue mitochondria isolation reagent A with phenylmethylsulfonyl fluoride (PMSF) was added, and then homogenized in an ice bath approximately 10 times. The homogenate was centrifuged at 600 rpm at 4 °C for 5 min. The supernatant was then collected and centrifuged at 11000 *g* at 4 °C for 10 min. The precipitate contained the mitochondria. The mitochondrial fractions of the lysate were used to detect Bax and Bcl-2 by Western blotting. Liver tissues were homogenized in RIPA Lysis Buffer with PMSF and then centrifuged at 12000 *g* for 15 min at 4 °C, and the supernatant contained the total protein.

### Western blot analysis

Cells or liver tissues were lysed on ice in lysis buffer (50 mM Tris-HCl [pH 8.0], 150 mM NaCl, 1% NP40, 0.5% sodium deoxycholate, 0.1% SDS, and 1 mM PMSF). Samples were fractionated by SDS-PAGE and transferred onto polyvinylidene fluoride (PVDF) membranes (Millipore, Billerica, MA, USA). Membranes were blocked in 5 % nonfat milk and 0.05 % Tween-20 in PBS for 2 h at room temperature and then incubated with a mouse anti-fgl2 antibody (1:750 dilution; Abnova, Taiwan, China), mFas (1:1000 dilution; Abcam, Cambridge, UK), mTNFR1 (1:1000 dilution; Abcam, Cambridge, UK), anti-c-IAP1, anti-Bcl-2, anti-Bax, anti-cleaved caspase-3 (Cell Signaling Technology, Beverly, MA) or anti-Bad (Abcam, Cambridge, MA). The membranes were then incubated with a goat anti-mouse or anti-rabbit HRP-labeled secondary antibody (1:5000 dilution; Santa Cruz Biotechnology, Santa Cruz, CA, USA). Protein bands were detected using enhanced chemiluminescence (ECL; Pierce, Rockford, IL, USA).

### Terminal deoxynucleotidyl transferase-mediated deoxyuridine 5-triphosphate nick end labeling assay

A terminal deoxynucleotidyl transferase (TdT)-mediated dUTP nick-end labeling (TUNEL) kit (Boster, Wuhan, China) was used to detect apoptosis in hepatocytes. Liver slices were deparaffinized in xylene and rehydrated through decreasing concentrations of ethanol. After incubation with 20 mg/ml proteinase K and 0.3% H_2_O_2_, the sections were washed with PBS. TdT and digoxin–dUTP were added overnight at 4°C, followed by incubation for 30 minutes with a biotinylated anti-digoxin antibody at 37°C and then bound with streptavidin–peroxidase. The slices were then washed with PBS, and color development was subsequently carried out using DAB. Hepatocytes with condensed brown particles within the nucleus were considered to have undergone apoptosis. 

### Statistical analysis

Quantitative data are expressed as mean ± standard error. Statistical significance was determined by variance analysis. All statistical analysis was carried out using SPSS v15.0 statistical software (SPSS, Chicago, IL, USA), and *P* values for significance were set at 0.05. 

## Results

### miRNA expression plasmids inhibit target gene expression in CHO cells

**Figure 1:**
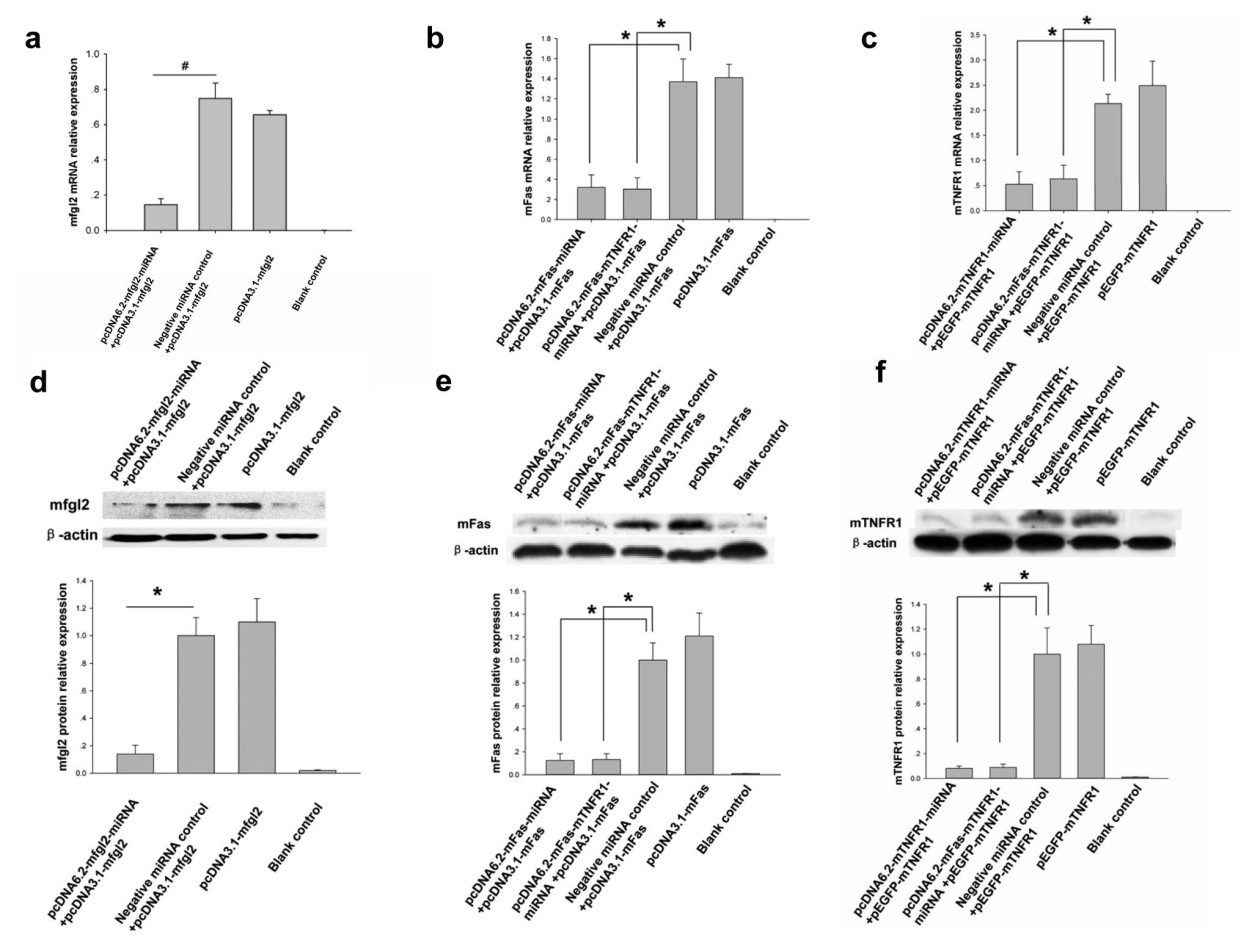
The constructed miRNA expression plasmids significantly inhibited target gene expression in CHO cells. (**a**-**c**) qRT-PCR showed that the miRNA eukaryotic expression plasmids targeting mfgl2, mFas, and mTNFR1, pcDNA6.2-mfgl2-miRNA (**a**), both pcDNA6.2-mFas-miRNA and pcDNA6.2-mFas-mTNFR1-miRNA (**b**), and both pcDNA6.2-mTNFR1-miRNA and pcDNA6.2-mFas-mTNFR1-miRNA (**c**), respectively, inhibited mfgl2, mFas, and mTNFR1 mRNA expression. Negative miRNA control: irrelevant miRNA plasmid, Blank control: CHO cells not treated. *P <0.05, #P <0.01 compared with the negative miRNA control group. (**d**-**f**) Western blot analysis showing that pcDNA6.2-mfgl2-miRNA (**d**), both pcDNA6.2-mFas-miRNA and pcDNA6.2-mFas-mTNFR1-miRNA (**e**), and both pcDNA6.2-mTNFR1-miRNA and pcDNA6.2-mFas-mTNFR1-miRNA (**f**) inhibited mfgl2, mFas, and mTNFR1 protein expression, respectively. The average protein expression from Negative miRNA control group was designated as 1. *P <0.05.

miRNA eukaryotic expression plasmids targeting mfgl2, mFas, and mTNFR1 (pcDNA6.2-mfgl2-miRNA, pcDNA6.2-mFas-miRNA, and pcDNA6.2-mTNFR1-miRNA) were constructed and confirmed by sequence analysis (data not shown). A mFas expression plasmid (pcDNA3.1-mFas) was also constructed and confirmed by PCR, enzyme restriction (Figure S3A), and sequence analysis (data not shown). Studies were undertaken to determine if the constructed miRNA plasmids expressing DNA code for non-native miRNA could interfere with target gene expression in a cell culture system. qRT-PCR indicated that pcDNA6.2-mfgl2-miRNA, pcDNA6.2-mFas-miRNA, and pcDNA6.2-mTNFR1-miRNA significantly inhibited mfgl2, mFas, and mTNFR1 mRNA expression, respectively. Compared with negative control, their inhibition efficiency reached 78%, 80%, and 73% in CHO, respectively (Figure 1 a, b, c). To further confirm that the inhibitory effects of these miRNA expression plasmids were also seen at the protein level, Western blot analysis was performed, and this showed inhibition of the respective target protein expression (Figure 1 d, e, f). Further experiments showed pcDNA6.2-mFas-miRNA and pcDNA6.2-mTNFR1-miRNA significantly inhibited mFas and mTNFR1 expression in mouse liver cell line IAR20 (Figure S4).

An expression plasmid that could express miRNA against both mFas and mTNFR1, named pcDNA6.2-mFas-mTNFR1-miRNA, was constructed and confirmed by restriction enzyme mapping (Figure S3B). Our experiments showed pcDNA6.2-mFas-mTNFR1-miRNA significantly inhibited mRNA and protein expression of both mFas and TNFR1 in CHO (Figure 1 b, c, e, f) and IAR20 (Figure S4). Based on the above results, we selected these artificial miRNAs to further construct adenovirus expression vectors for *in vivo* interference.

### Construction of Ad-mfgl2-miRNA and Ad-mFas-mTNFR1-miRNA

pcDNA6.2-mfgl2-miRNA was cloned into pAd/CMV/V5-DEST using Gateway technology, then the resulting plasmid was transfected into 293A cells to generate a replication-deficient recombinant adenovirus encoding an artificial miRNA against mfgl2 (Ad-mfgl2-miRNA). Using the same procedure, Ad-mFas-mTNFR1-miRNA was constructed from pcDNA6.2-mFas-mTNFR1-miRNA. A negative miRNA control adenovirus containing an irrelevant sequence pre-miRNA was also constructed and was predicted not to target on any known vertebrate gene. Ad-mfgl2-miRNA and Ad-mFas-mTNFR1-miRNA were successfully constructed, as evidenced by infected 293A cells containing viral particles expressing green fluorescence (Figure S3C).

### Combined interference resulted in reduced lethality, improved liver function and pathology in fulminant hepatitis

**Figure 2:**
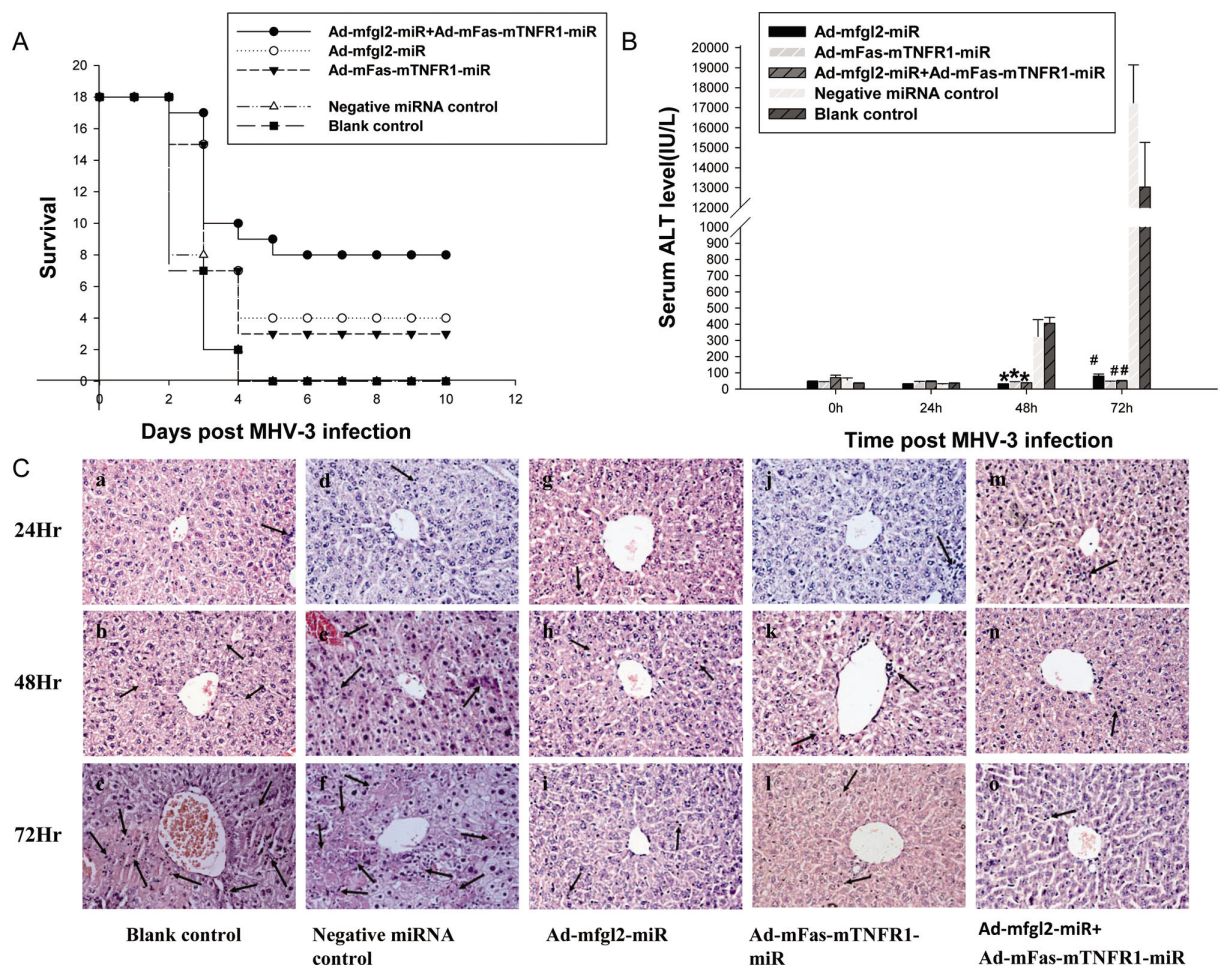
Combined interference increased the survival rate and improved liver function and histopathology in mice. (**A**): Combined interference with Ad-mfgl2-miRNA and Ad-mFas-mTNFR1-miRNA had a higher survival rate than that of interference with either construct alone in MHV-3–infected BALB/cJ mice. Ad-mfgl2-miRNA, Ad-mFas-mTNFR1-miRNA, or irrelevant miRNA control adenovirus were introduced into BALB/cJ mice by tail vein injection. The mice then received 20 PFU of MHV-3 intraperitoneally 24 hours later to promote the development of fulminant viral hepatitis. Survival data are presented. Serial serum ALT levels (**B**) and histopathology (**C**) (H&E staining; original magnification, ×400) at 24, 48, and 72 h post MHV-3 infection were evaluated in the five groups of mice. (**B**) Effect of Ad-mfgl2-miRNA and/or Ad-mFas-mTNFR1-miRNA on serum ALT levels. Values represent means and standard error of three independent experiments done in triplicate. *P <0.05, #P <0.01 compared with the negative miRNA control adenovirus group. (**C**) Effect of Ad-mfgl2-miRNA and/or Ad-mFas-mTNFR1-miRNA on liver histopathology in MHV-3–infected BALB/cJ mice. Livers were collected from Ad-mfgl2-miRNA–treated (**g**, **h**, and **i**), Ad-mFas-mTNFR1-miRNA–treated (**j**, **k**, and **l**), combined interference-treated (**m**, **n**, and **o**), irrelevant miRNA adenovirus-treated (**d**, **e**, and **f**), or PBS-treated (**a**, **b**, and **c**) BALB/cJ mice at 24 h (a, d, g, j, and m), 48 h (b, e, h, k, and n), and 72 h (c, f, i, l, and o) after MHV-3 infection. Arrows point to inflammatory cell infiltration areas or necrotic areas with the inflammation.

To investigate the biological effect of Ad-mfgl2-miRNA and Ad-mFas-mTNFR1-miRNA *in vivo*, single or combined adenovirus expression vectors were delivered by tail vein injection into MHV-3-infected BALB/cJ mice (18 mice/group). In the combined interference group (Ad-mfgl2-miRNA and Ad-mFas-mTNFR1-miRNA) 17 mice were alive on day 3 after infection, 10 mice alive on day 4, and 8 of 18 (44.4%) mice recovered from fulminant viral hepatitis. At the same time, in either Ad-mfgl2-miRNA or Ad-mFas-mTNFR1-miRNA treated group, there were 15 mice alive on day 3 and 7 mice alive on day 4 after infection in each group. 4 of 18 (22.2%) mice receiving Ad-mfgl2-miRNA treatment alone recovered, and 3 of 18 (16.7%) mice treated with Ad-mFas-mTNFR1-miRNA were alive on day 10 post infection. In contrast, in the negative miRNA control adenovirus-treated group and the blank control group, 8 and 7 mice were alive on day 3 after infection, respectively, only 2 mice alive on day 4 in each group, and no mice survived to day 5 (Figure 2A).

To better understand the mechanism underlying the biological effects of combined interference with Ad-mfgl2-miRNA and Ad-mFas-mTNFR1-miRNA, a time-course study of liver function (serum ALT levels) and liver histology in MHV-3-infected mice was performed. The serum ALT levels significantly increased at 48 h after infection in both negative control and blank control groups. However, at 48 h and 72 h after infection, significant increase of serum ALT was not observed in the targeted gene interference mice, particularly in the combined interference mice, when compared with the negative control group (Figure 2B).

Liver histology was examined by hematoxylin and eosin (H&E) staining at the indicated time points. There were rarely inflammatory infiltration and hepatocyte necrosis in the livers of the five groups 24 h after MHV-3 infection (Figure 2C, panels a, d, g, j, and m). At 48 h and 72 h after MHV-3 infection, only mild inflammatory cell infiltration, with no evidence of hepatocyte necrosis, was found in the combined interference mice (Figure 2C, panel n and o) compared with the focal and evenly distributed necrosis and massive inflammatory cell infiltration in both negative control (Figure 2C, panel e and f) and untreated mice (Figure 2C, panel b and c). Gene interference with either Ad-mfgl2-miRNA (Figure 2C, panel h and i) or Ad-mFas-mTNFR1-miRNA (Figure 2C, panel k and l) also markedly ameliorated hepatic inflammatory infiltration and hepatocyte necrosis. These data strongly suggested that combined interference with Ad-mfgl2-miRNA and Ad-mFas-mTNFR1-miRNA dramatically improved liver function and pathology in mice with MHV-3-induced fulminant viral hepatitis.

The effect of constructed miR adenovirus expression plasmid on the virus level in hepatocytes of infected mice was investigated. Our results showed that, at 24h, 48h and 72h after MHV-3 injection, the virus proliferated significantly in all five groups, but there was no statistical difference among them at each time point (Figure S5).

The effect of adenoviral constructs on cardiac tissue and liver was further investigated. Our results showed there were rarely infiltrating inflammatory cells and tissue necrosis in heart and liver till 24 days after injection of adenoviral constructs. (Figure S6).

### Effect of Ad-mfgl2-miRNA and Ad-mFas-mTNFR1-miRNA on target gene expression and related fibrin deposition and intrahepatic apoptosis in MHV-3-infected mice

**Figure 3:**
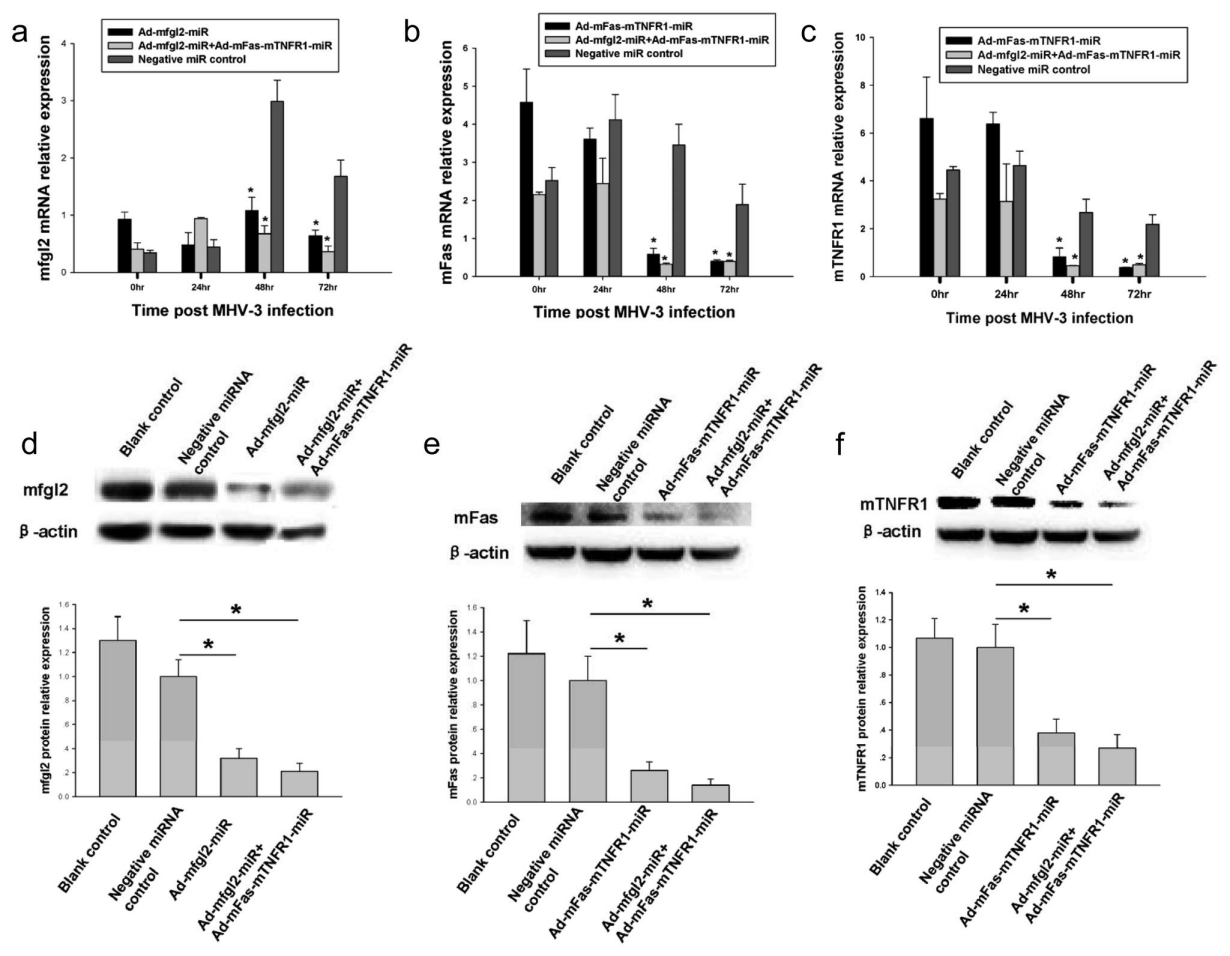
Ad-mfgl2-miRNA and/or Ad-mFas-mTNFR1-miRNA inhibited target genes at both mRNA and protein levels *in* *vivo*. The treatment process was the same as that described in Figure 2, and livers were collected from treated BALB/cJ mice 0, 24, 48, and 72 h after MHV-3 infection. (**a**) qRT-PCR showed both Ad-mfgl2-miRNA and combined interference with Ad-mfgl2-miRNA and Ad-mFas-mTNFR1-miRNA inhibited mfgl2 mRNA expression 48 h and 72 h after MHV-3 infection. Both Ad-mFas-mTNFR1-miRNA and combined interference with Ad-mfgl2-miRNA and Ad-mFas-mTNFR1-miRNA inhibited mFas (**b**) and mTNFR1 (**c**) mRNA expression 48 h and 72 h after MHV-3 infection also. Values represent means and SE of three separate experiments done in triplicate.*P <0.05 compared with Negative miRNA control adenovirus group. (**d**-**f**) Western blot analysis showed combined interference with Ad-mfgl2-miRNA and Ad-mFas-mTNFR1-miRNA inhibited mfgl2 (**d**), mFas (**e**), and mTNFR1 (**f**) protein expression at 72 h after MHV-3 infection. The average protein expression from Negative miRNA control group was designated as 1. *P <0.05.

To evaluate the inhibitory effects of Ad-mfgl2-miRNA and Ad-mFas-mTNFR1-miRNA *in vivo*, a time course study was performed via real-time fluorescence quantitative PCR using liver samples from mice at 0, 24, 48, and 72 h after MHV-3 infection. The inhibitory effect of Ad-mfgl2-miRNA or Ad-mFas-mTNFR1-miRNA began at 48 h after MHV-3 infection, with a maximal inhibitory effect at 72 h post infection (Figure 3 a, b, and c). Combined interference with Ad-mfgl2-miRNA and Ad-mFas-mTNFR1-miRNA significantly inhibited mfgl2 expression as well as mFas and mTNFR1 expression at both 48 and 72 h after MHV-3 infection (Figure 3 a, b, and c). These results were confirmed by Western blot analysis with specific monoclonal antibodies against mfgl2, mFas, and mTNFR1, respectively. The protein expression of mfgl2 (Figure 3 d), mFas (Figure 3 e), and mTNFR1 (Figure 3 f) decreased significantly in miRNA treated mice, including both single interference group and combined interference group. 

**Figure 4:**
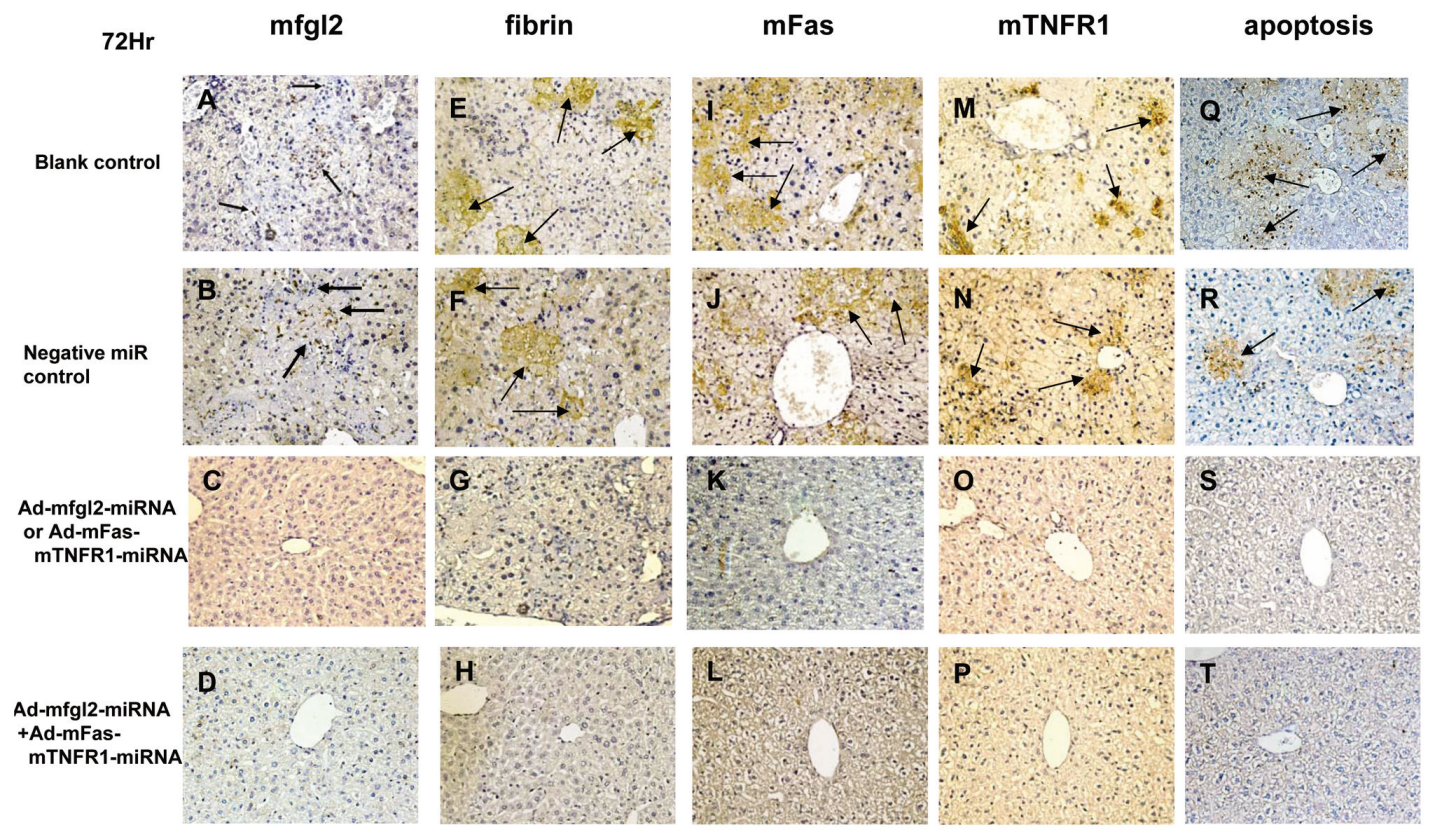
Combined interference inhibited target gene expression and decreased fibrin deposition and intrahepatic apoptosis in mice. Livers from BALB/cJ mice treated with either Ad-mfgl2-miRNA or Ad-mFas-mTNFR1-miRNA, from combination interference mice treated with Ad-mfgl2-miRNA and Ad-mFas-mTNFR1-miRNA, and from control-treated mice were collected 72 h after MHV-3 infection. mfgl2 expression (**A**–**D**), fibrin deposition (**E**–**H**), mFas expression (**I**–**L**), mTNFR1 expression (**M**–**P**) were determined by immunohistochemistry analysis, and hepatocyte apoptosis (**O**–**T**) was determined by TUNEL. Arrows represent positive staining as shown in brown. Original magnification, ×400.

**Figure 5:**
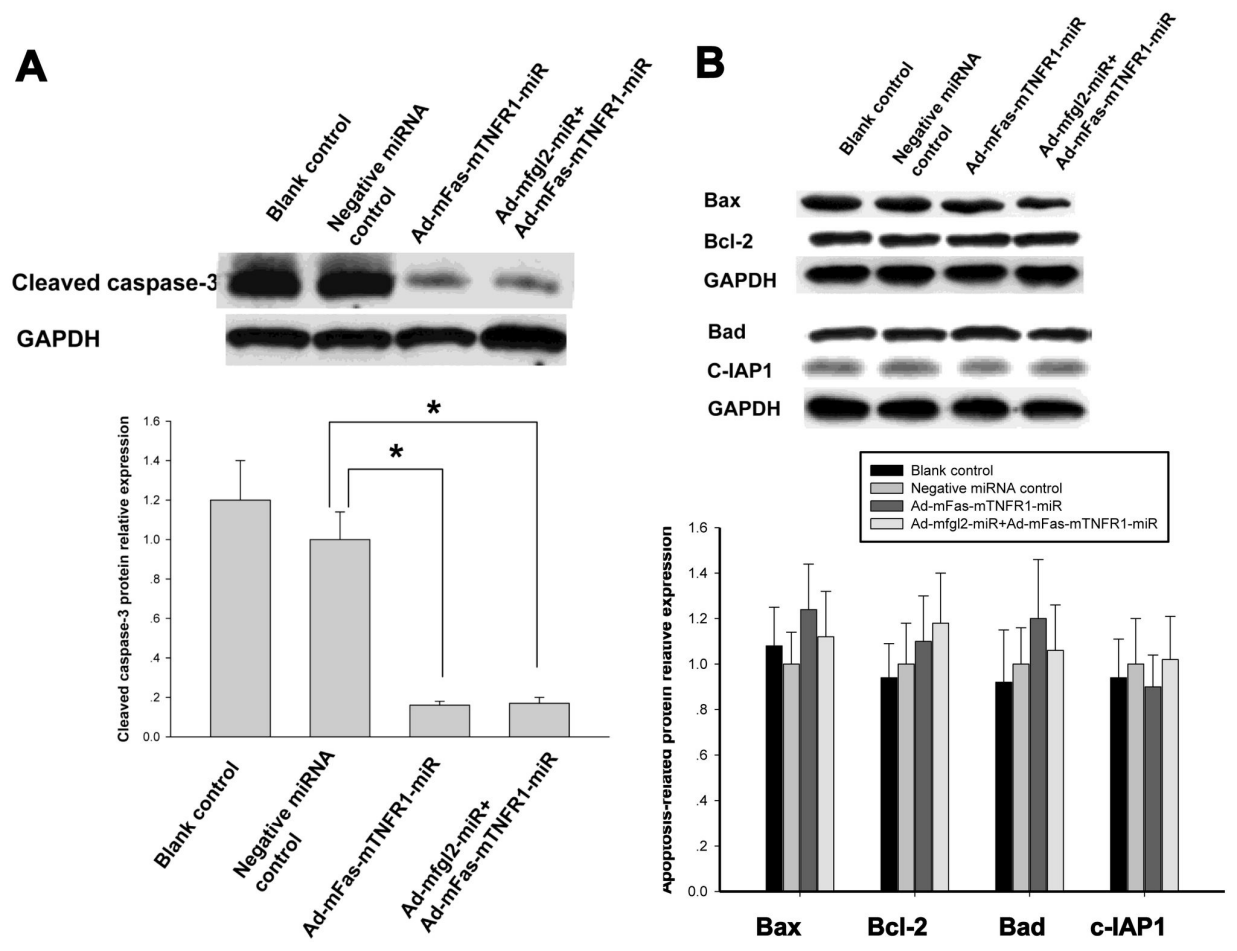
The constructed miR adenovirus did not affect the expression of certain apoptosis-related proteins, but significantly decreased cleavage of caspase-3. (**A**) There was significantly decreased cleavage of caspase-3 in Ad-mFas-mTNFR1-miRNA treated mice and combined interference group at 72 h after MHV-3 infection. (**B**) Both Ad-mFas-mTNFR1-miR and combined interference with the two adenoviral miRNAs did not affect the expression of pro-apoptotic proteins, including Bax and Bad, and anti-apoptotic proteins, including Bcl-2 and c-IAP2 at 72 h after MHV-3 infection. The average protein expression from Negative miRNA control group was designated as 1. *P <0.05.

These results were further confirmed by immunohistochemistry with polyclonal antibodies against mfgl2, mFas, and mTNFR1, respectively. Rare hepatic mfgl2 expression in Ad-mfgl2-miRNA-treated mice (Figure 4C), and no mfgl2 expression in the combined interference group (Figure 4D) were evidenced, along with a significant decrease of fibrin deposition in the liver tissues from mice treated with either mono (Figure 4G) or combined interference (Figure 4H), compared with that of mice treated with the negative (Figure 4B, F) or blank control (Figure 4A, E). Meanwhile, rare mFas or mTNFR1 expression in Ad-mFas-mTNFR1-miRNA treated mice (Figure 4K, O), and no mFas or mTNFR1 expression in the combined interference group (Figure 4L, P) were evidenced, accompanied with significantly decreased hepatocellular apoptosis (Figure 4S, T), compared with that of mice treated with the negative (Figure 4J, N, R) or blank controls (Figure 4I, M, Q). Further experiment showed there was significantly decreased cleavage of caspase-3 in Ad-mFas-mTNFR1-miRNA treated mice and combined interference group (Figure 5A).

Moreover, both Ad-mFas-mTNFR1-miR and combined interference with the two adenoviral miRNAs did not affect the expression of certain apoptosis-related proteins, such as pro-apoptotic proteins, including Bax and Bad, and anti-apoptotic proteins, including Bcl-2 and c-IAP2 (Figure 5B).

## Discussion

Patients with chronic HBV infection are at a significantly increased risk for the development of active chronic hepatitis B with severe acute exacerbations or flares that may lead to liver failure and mortality [21,22]. Management strategies have been developed for ALF during recent decades such as liver support systems, however, meta-analysis has shown that liver support systems have no significant survival benefit in patients with liver failure when compared with standard medical therapy. Liver transplantation is now the most promising treatment for ALF in many countries for those patients who meet the criteria indicative of a poor prognosis. However, it is feasible in only a minority of patients because of the shortage of donor organs. 

As such, efforts have been dedicated to investigating the related immune-mediated liver injury and regulating the imbalanced in the immune system. Many studies have demonstrated that patients with HBV-ACLF undergo an excessive immune response to HBV, displayed as massive necrosis and apoptosis [5,23]. Activated endothelial cells and macrophages express a distinct cell-surface procoagulant, a novel prothrombinase Fgl2/fibroleukin, which is a member of the fibrinogen family of proteins and is an immune coagulant with the ability to directly catalyze prothrombin to thrombin in the absence of factor VII or factor X [24,25]. Fgl2 prothrombinase is known to play a critical role in the hepatocellular injury observed in both MHV-3-induced fulminant hepatitis and human severe hepatitis B. Thus, human Fgl2/fibroleukin (hfgl2) was proposed as a target for therapeutic intervention [26]. 

Hepatocyte apoptosis in the liver plays an important role in the processes of many liver diseases, particularly hepatic failure resulting from various causes. In the process of severe hepatitis, activation of Fas and TNF affects the severity of hepatocyte apoptosis [27]. Many studies have revealed that the FasL/Fas system is involved in massive hepatocyte destruction in patients with ALF [28-31]. Fas is constitutively expressed on hepatocyte cell membranes and is activated when bound to membrane-bound FasL or soluble FasL [32]. In MHV-3–

infected BALB/cJ mice, the expression of FasL is highly upregulated on liver NK cells at 48 h post infection, meanwhile Fas is increased on hepatocytes post infection and the FasL/Fas pathway contributes to NK cell-induced hepatocyte injury in MHV-3–induced fulminant hepatic failure [5]. In human ALF, hepatocytes strongly express Fas, FasL is upregulated on infiltrating lymphocytes, and serum soluble FasL is markedly elevated [33]. 

TNF-induced hepatocyte apoptosis may be of major importance under conditions of ALF in septic shock [34]. The pathogenic role of TNFα has been extensively documented in both experimental models and human ALF [35]. Hepatic failure and tissue destruction as a result of endogenously produced TNF are mediated by the 55-kDa TNFR1 [36]. In patients with ALF, TNFR1 was found to be significantly increased on hepatocytes, and so was serum level of TNF-α and TNFR1[37,38]. Results from our previous study showed that dual interference with shRNA expression plasmids targeting mfgl2 and mTNFR1 ameliorated MHV-3-induced fulminant hepatitis in BALB/cJ mice [39]. In this study, *in vivo* interference with Ad-mFas-mTNFR1-miRNA or combination with both Ad-mfgl2-miRNA and Ad-mFas-mTNFR1-miRNA efficiently silenced mFas and mTNFR1 gene expression, and there was no effect on the expression of other apoptosis-related proteins. In view of their vital role in severe hepatitis, Fas and TNFR1 could also be potential interference targets.

RNA interference (RNAi) is a natural process by which small dsRNA molecules direct sequence-specific post-transcriptional silencing of homologous genes by binding to complementary mRNAs and triggering their elimination [40]. The successful use of chemically synthesized small interfering RNAs (siRNAs), plasmid expression of small hairpin RNAs (shRNAs), or plasmid expression of miRNAs to silence target genes makes RNAi a powerful tool for studying gene function and may be a rational therapeutic approach for a variety of diseases. However, several challenges that need to be overcome include poor siRNA stability, inefficient cellular uptake, widespread biodistribution, and non-specific effects. In the present study, an irrelevant miRNA plasmid was constructed to test the specificity of the miRNA expression plasmids, and the results showed it failed to inhibit gene expression at the level of either transcription or translation. Further investigation showed the proliferation of MHV-3 was unaffected by these adenoviral constructs. These data provided evidence of the specificity of the miRNA adenovirus expression vectors.

It is critical for gene therapy that a suitable vector is used *in vivo*. Due to the short half-life, low *in vivo* transfection efficiency, and necessary assistance by hydrodynamic injection, plasmid-based shRNAs are unlikely to be adapted for use in primates. To overcome these limitations, adenoviral vectors are being developed. Replication deficient-recombinant adenovirus is one of the most advanced and best-studied vector systems capable of expressing exogenous genes at a high level [41]. They are highly efficient at transferring genes to a wide variety of target cells, including both dividing and nondividing cells. More importantly, when administered intravenously at a high titer, over 95% of hepatocytes have been shown to be transduced with adenovirus [42,43]. Peak expression of adenovirus-

mediated transgenes occurs 2~7 days after adenoviral administration [44]. So it is considered that adenovirus vectors are suitable for treatment of acute liver failure, which is the clinical manifestation of sudden and severe hepatic injury. 

One emerging concern is that RNAi mono-therapy might ultimately fail to control disease progress. Thus, sophisticated strategies are being developed that aim at combining RNAi effectors with each other. Two therapeutic genes, suppressor of cytokine signaling 3 and tumor necrosis factor-related apoptosis inducing ligand were incorporated into an oncolytic adenovirus vector, and combined treatment with these exhibited potent antitumor activity compared with any other treatment [45]. Co-expression of two tumor suppressor genes, interleukine-24 and shRNA against M-phase phosphoprotein 1, in one adenoviral vector significantly raised the anti-tumor effect [46]. Our prior report has validated this new concept of combinatorial RNAi and illustrated its versatility by co-expression of shRNAs against two targets [39]. The ability to express multiple miRNAs is important to our overall strategy since it helps to broaden the interference efficacy against ALF, which is a syndrome of diverse etiologies. 

In the current study, we used type-5 recombinant adenoviral vectors with deletion of the adenoviral early genes E1 and E3. Considering fgl2 prothrombinase was mainly expressed on activated endothelial cells and macrophages, meanwhile, Fas and TNFR1 were constitutively expressed on hepatocytes, we constructed adenoviral vectors carrying DNA code for non-native miRNAs that targeted mfgl2 or targeted both mFas and mTNFR1, respectively. Our study showed that adenoviral gene transfer of artificial miRNAs against immune-related factors, single or combined use, protected BALB/cJ mice from MHV-3–induced fulminant hepatitis, by significantly ameliorating inflammatory infiltration, fibrin deposition, hepatocyte necrosis, and apoptosis. More importantly, these miRNAs also prolonged survival time and increased the survival rate of the mice, especially the combined interference.

In conclusion, our data showed that combined interference with recombinant adenoviral vectors carrying artificial miRNAs against mfgl2, mFas, and mTNFR1 might have a synergistic effect and have significant therapeutic potential for the treatment of fulminant hepatitis. It could become a new and different management approach to ALF and make a contribution to the treatment of this disease. Our results with this efficient knockdown system may extend our knowledge of gene therapy. Since this method has only been successful in laboratory studies, it remains a challenge to deliver RNAi agents into liver cells in a manner that will be clinically relevant in patients. As such, preclinical tests are worthy of further study. 

## Supporting Information

Figure S1 **A sketch of construction of miRNA expression plasmids.**
(TIF)

Figure S2 **A sketch of construction of pcDNA6.2-mFas-mTNFR1-miRNA.**
(TIF)

Figure S3 **Construction of pcDNA3.1-mFas, Ad-mfgl2-miRNA and Ad-mFas-mTNFR1-miRNA.** (**A**) Construction of pcDNA3.1-mFas was confirmed by PCR and enzyme restriction. Lane 1: PCR product; Lane 2: enzyme restriction map with *Hin*dIII and *Bam*HI; Lane 3: pcDNA3.1-mFas; M: DNA marker. (**B**) The construction of pcDNA6.2-mFas-mTNFR1-miRNA was verified with *Bam*HI and *Bgl*II. Lane 1: enzyme restriction map of pcDNA6.2-mFas-mTNFR1-miRNA. Lane 2: enzyme restriction map of pcDNA6.2-mFas-miRNA. Lane 3: DNA marker. (**C**) Adenovirus-infected 293A cells were evidenced by fluorescence microscopy. a: Ad-mfgl2-miRNA. b: Ad-mFas-mTNFR1-miRNA. Original magnification, ×200.
(TIF)

Figure S4 **The constructed miRNA expression plasmids significantly inhibited target gene expression in mouse liver cell line IAR20.** (**a**, **b**) qRT-PCR showed that the miRNA eukaryotic expression plasmids targeting mFas and mTNFR1, both pcDNA6.2-mFas-miRNA and pcDNA6.2-mFas-mTNFR1-miRNA (**a**), and both pcDNA6.2-mTNFR1-miRNA and pcDNA6.2-mFas-mTNFR1-miRNA (**b**), respectively, inhibited mFas and mTNFR1 mRNA expression. Negative miRNA control: irrelevant miRNA plasmid, Blank control: IAR20 cells not treated. *P <0.05, compared with the negative miRNA control group. (**c**, **d**) Western blot analysis showing that both pcDNA6.2-mFas-miRNA and pcDNA6.2-mFas-mTNFR1-miRNA (**c**), and both pcDNA6.2-mTNFR1-miRNA and pcDNA6.2-mFas-mTNFR1-miRNA (**d**) inhibited mFas and mTNFR1 protein expression, respectively. The average protein expression from Negative miRNA control group was designated as 1. *P <0.05.
(TIF)

Figure S5 **The effect of constructed miR adenoviral expression plasmid on the levels of MHV-3 in hepatocytes of infected mice.** Livers were collected from different treated BALB/cJ mice at 24 h, 48 h, and 72 h after MHV-3 infection.
(TIF)

Figure S6 **The effect of adenoviral constructs on cardiac tissue and liver.** Tissue was collected on 1, 3, 7, 11 and 24 days post MHV-3 infection. H&E staining, original magnification, ×400.
(TIF)
