# Supplementary figures and images for: Combined Adenovirus-Mediated Artificial microRNAs Targeting mfgl2, mFas, and mTNFR1 Protect against Fulminant Hepatic Failure in Mice
Source: PLoS One. 2013 Nov 26;8(11):e82330. doi: 10.1371/journal.pone.0082330 (PMC3841162; doi:10.1371/journal.pone.0082330)

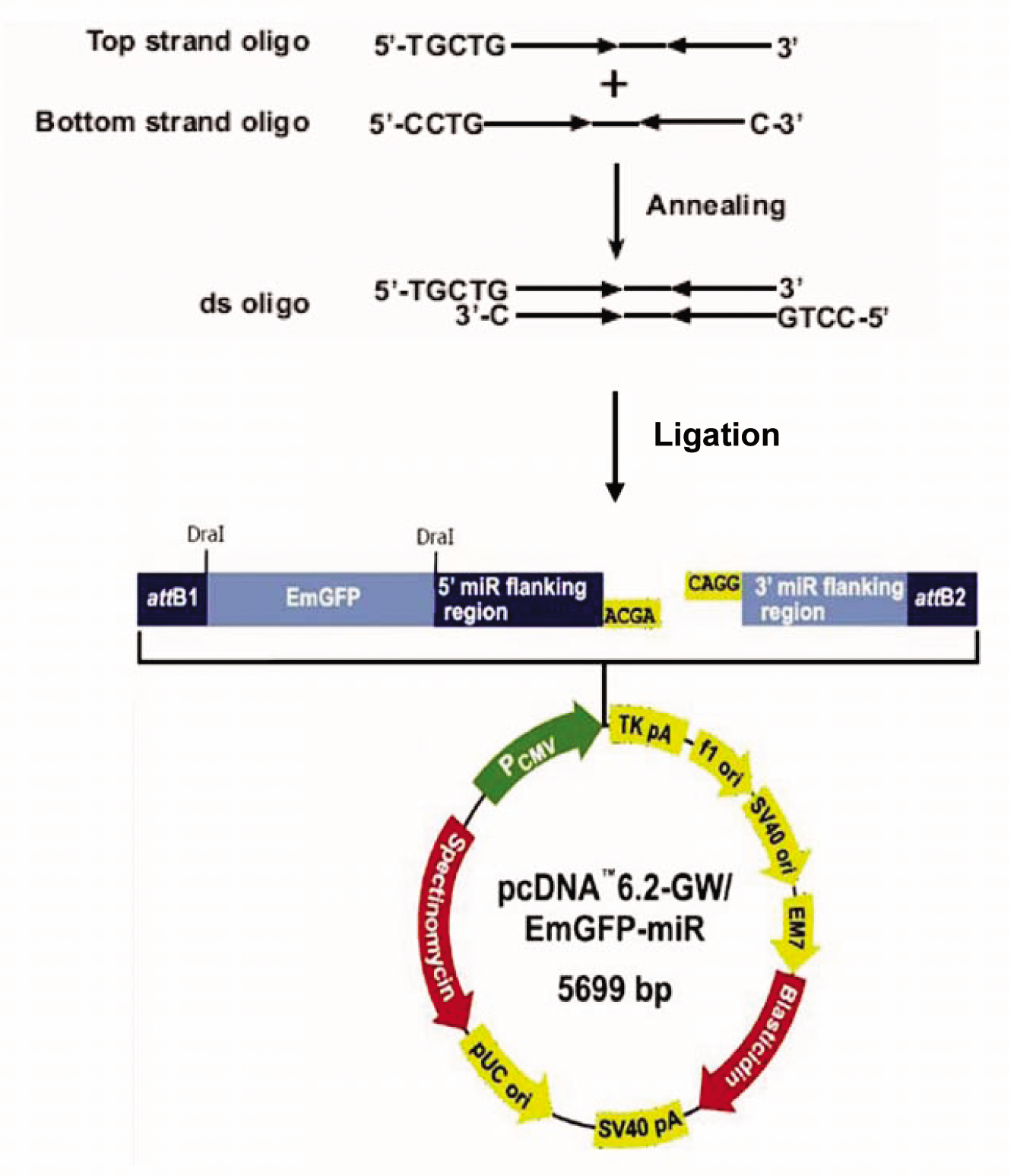

Supplement: Figure S1 — A sketch of construction of miRNA expression plasmids. (TIF) [file pone.0082330.s001.tif]

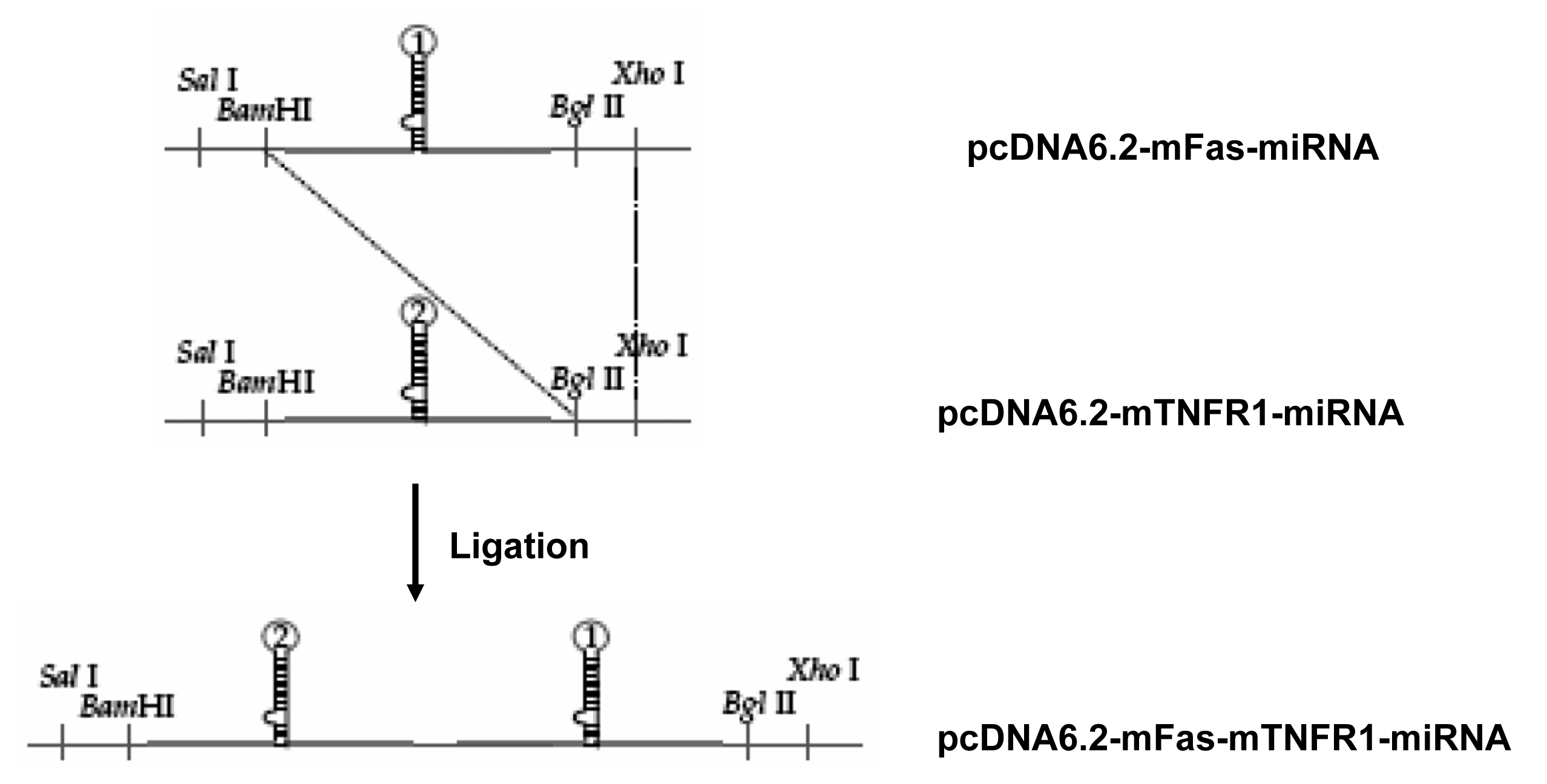

Supplement: Figure S2 — A sketch of construction of pcDNA6.2-mFas-mTNFR1-miRNA. (TIF) [file pone.0082330.s002.tif]

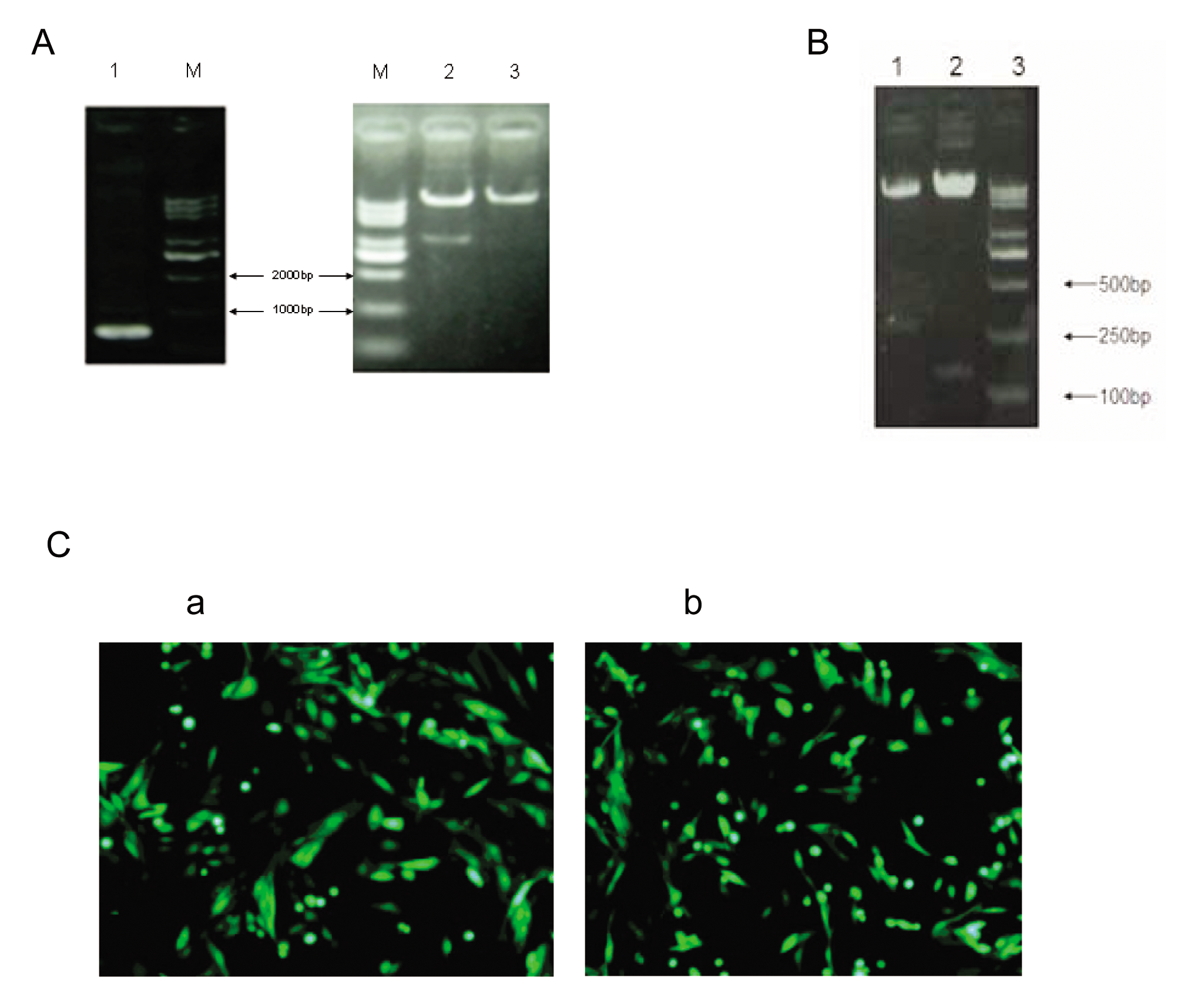

Supplement: Figure S3 — Construction of pcDNA3.1-mFas, Ad-mfgl2-miRNA and Ad-mFas-mTNFR1-miRNA. (A) Construction of pcDNA3.1-mFas was confirmed by PCR and enzyme restriction. Lane 1: PCR product; Lane 2: enzyme restriction map with HindIII and BamHI; Lane 3: pcDNA3.1-mFas; M: DNA marker. (B) The construction of pcDNA6.2-mFas-mTNFR1-miRNA was verified with BamHI and BglII. Lane 1: enzyme restriction map of pcDNA6.2-mFas-mTNFR1-miRNA. Lane 2: enzyme restriction map of pcDNA6.2-mFas-miRNA. Lane 3: DNA marker. (C) Adenovirus-infected 293A cells were evidenced by fluorescence microscopy. a: Ad-mfgl2-miRNA. b: Ad-mFas-mTNFR1-miRNA. Original magnification, ×200. (TIF) [file pone.0082330.s003.tif]

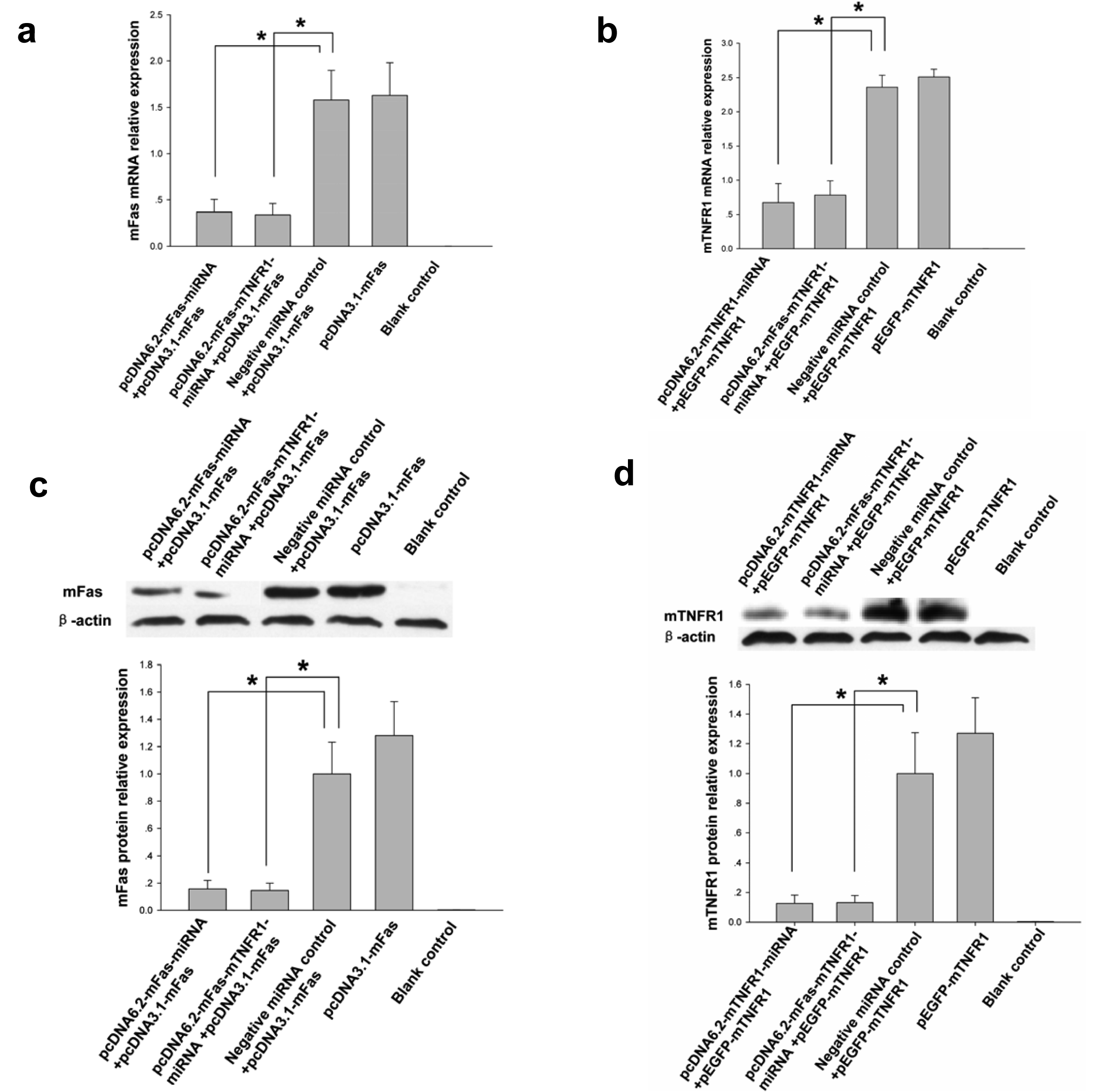

Supplement: Figure S4 — The constructed miRNA expression plasmids significantly inhibited target gene expression in mouse liver cell line IAR20. (a, b) qRT-PCR showed that the miRNA eukaryotic expression plasmids targeting mFas and mTNFR1, both pcDNA6.2-mFas-miRNA and pcDNA6.2-mFas-mTNFR1-miRNA (a), and both pcDNA6.2-mTNFR1-miRNA and pcDNA6.2-mFas-mTNFR1-miRNA (b), respectively, inhibited mFas and mTNFR1 mRNA expression. Negative miRNA control: irrelevant miRNA plasmid, Blank control: IAR20 cells not treated. *P <0.05, compared with the negative miRNA control group. (c, d) Western blot analysis showing that both pcDNA6.2-mFas-miRNA and pcDNA6.2-mFas-mTNFR1-miRNA (c), and both pcDNA6.2-mTNFR1-miRNA and pcDNA6.2-mFas-mTNFR1-miRNA (d) inhibited mFas and mTNFR1 protein expression, respectively. The average protein expression from Negative miRNA control group was designated as 1. *P <0.05. (TIF) [file pone.0082330.s004.tif]

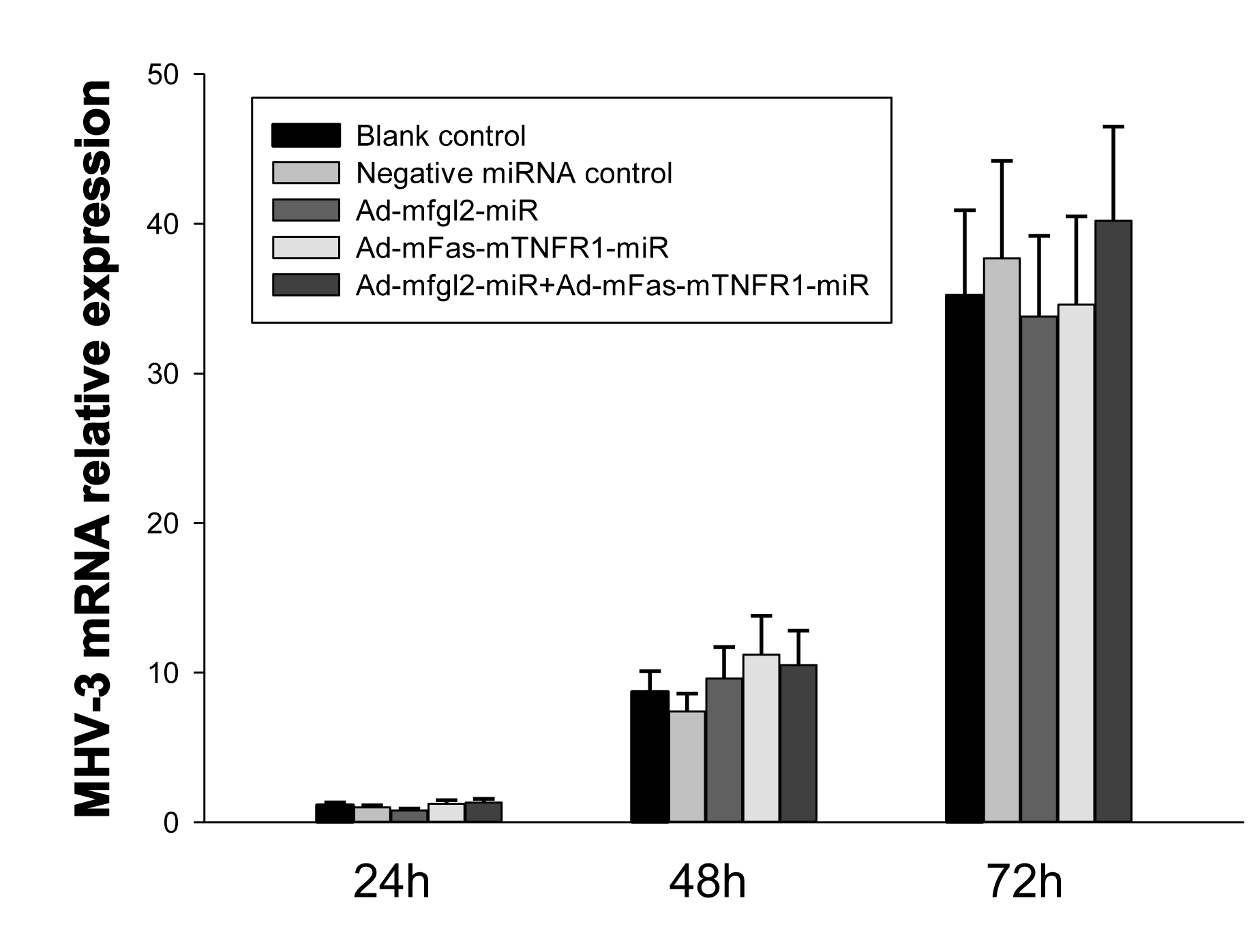

Supplement: Figure S5 — The effect of constructed miR adenoviral expression plasmid on the levels of MHV-3 in hepatocytes of infected mice. Livers were collected from different treated BALB/cJ mice at 24 h, 48 h, and 72 h after MHV-3 infection. (TIF) [file pone.0082330.s005.tif]

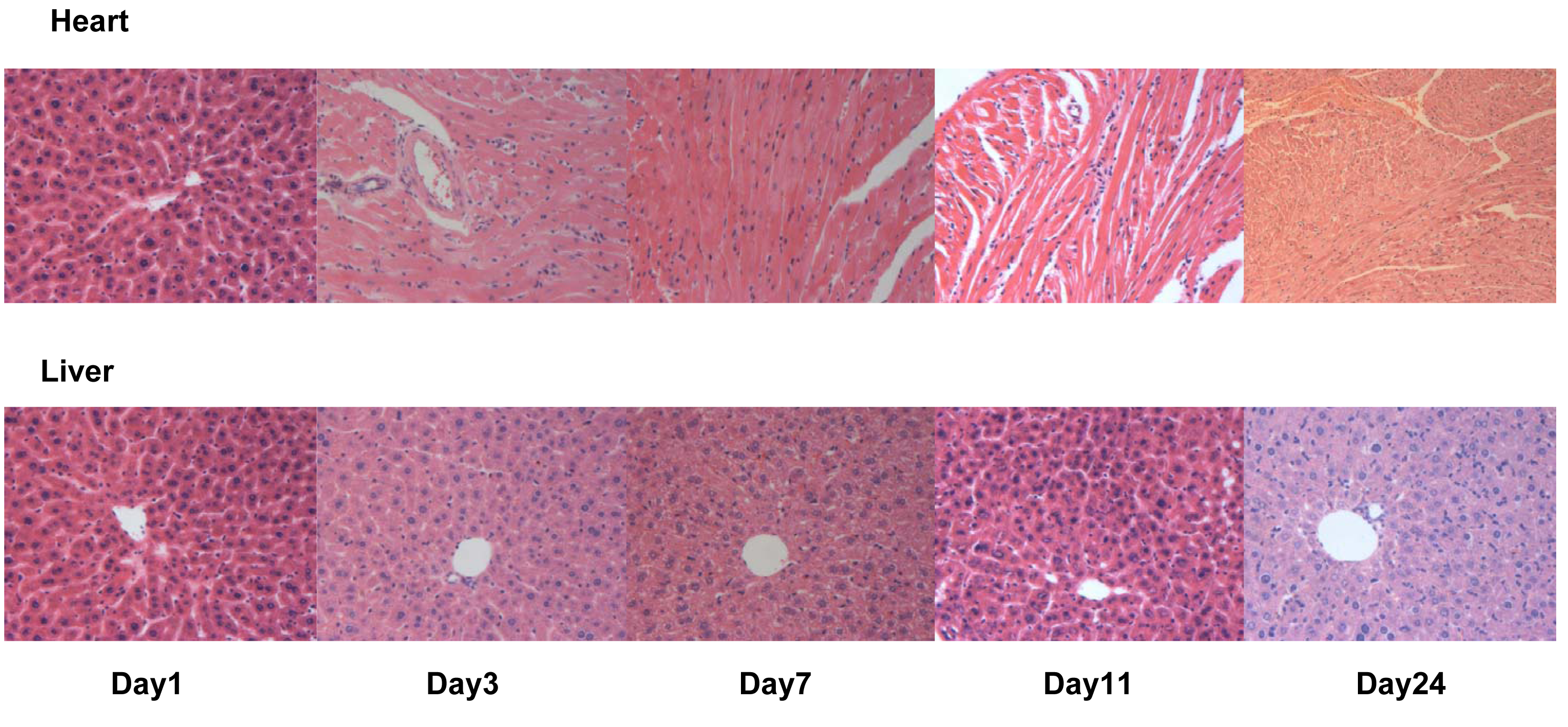

Supplement: Figure S6 — The effect of adenoviral constructs on cardiac tissue and liver. Tissue was collected on 1, 3, 7, 11 and 24 days post MHV-3 infection. H&E staining, original magnification, ×400. (TIF) [file pone.0082330.s006.tif]
